# Human pluripotent stem cell models of cardiac disease: from mechanisms to therapies

**DOI:** 10.1242/dmm.030320

**Published:** 2017-09-01

**Authors:** Karina O. Brandão, Viola A. Tabel, Douwe E. Atsma, Christine L. Mummery, Richard P. Davis

**Affiliations:** 1 Department of Anatomy and Embryology, Leiden University Medical Center, 2300 RC Leiden, The Netherlands; 2 Department of Cardiology, Leiden University Medical Center, 2300 RC Leiden, The Netherlands

**Keywords:** Cardiac arrhythmia, Cardiometabolic disease, Cardiomyopathy, Disease model, Genetic cardiac disease, Pluripotent stem cell, hiPSC

## Abstract

It is now a decade since human induced pluripotent stem cells (hiPSCs) were first described. The reprogramming of adult somatic cells to a pluripotent state has become a robust technology that has revolutionised our ability to study human diseases. Crucially, these cells capture all the genetic aspects of the patient from which they were derived. Combined with advances in generating the different cell types present in the human heart, this has opened up new avenues to study cardiac disease in humans and investigate novel therapeutic approaches to treat these pathologies. Here, we provide an overview of the current state of the field regarding the generation of cardiomyocytes from human pluripotent stem cells and methods to assess them functionally, an essential requirement when investigating disease and therapeutic outcomes. We critically evaluate whether treatments suggested by these *in vitro* models could be translated to clinical practice. Finally, we consider current shortcomings of these models and propose methods by which they could be further improved.

## Abstract (precis)

**Summary:** This Review discusses how human pluripotent stem cells have revolutionised the study of cardiac disease, highlighting their impact in investigating pathogenesis and their use in drug discovery, as well as current challenges facing the field.

## Introduction

Since the derivation of human embryonic stem cells (hESCs) was first described in 1998 (Thomson et al., 1998), there has been an expectation that this knowledge would usher in a new age of medicine, particularly for regenerative medicine. Although stem-cell-based therapies involving cell transplantation are becoming a reality for some diseases (Kimbrel and Lanza, 2015; Trounson and McDonald, 2015), for many conditions, including myocardial infarction (see Box 1 for a glossary of terms), significant hurdles still need to be overcome to bring such a treatment to the clinic. However, the more recent ability to generate human induced pluripotent stem cells (hiPSCs) from adult cells (Takahashi et al., 2007; Yu et al., 2007) has created new opportunities to study the mechanisms underlying human genetic diseases and, as a corollary, develop new therapeutic strategies.

One area of major interest in the hiPSC-based research arena is that of cardiac disease. The prevalence of cardiac disorders is increasing, partially owing to improved detection and survival of patients with inherited forms of such diseases, such as primary arrhythmias (Box 1), cardiomyopathies and cardiometabolic diseases. This, however, has not been met with a commensurate improvement in strategies to treat these disorders, in part due to the limitations of existing experimental models. Notably, although animal models have made a major contribution to our understanding of cardiovascular disease, interspecies differences at both a genetic and physiological level confound our ability to translate these findings to treatments for humans (Davis et al., 2011).

Inherited cardiovascular disorders were among the first diseases for which hiPSC lines were derived from patients (Bellin et al., 2012). Such models are not only providing insight into the pathogenesis of many inherited cardiac diseases, but are also being used to develop novel ways to treat them. In this Review, we summarise recent developments for efficiently differentiating both hESCs and hiPSCs [collectively referred to as human pluripotent stem cells (hPSCs)] to cardiomyocytes (cardiac muscle cells), and approaches for functional characterisation of these cells (Box 1). We provide an overview of the many hPSC-cardiomyocyte (hPSC-CM) models of inherited cardiac diseases that have been described to date and highlight how they have revealed novel disease mechanisms and therapeutic approaches for the pathologies. Finally, we comment on the current challenges faced by researchers using hPSC models to investigate cardiac diseases and provide our perspectives on possible solutions.

**Box 1. Glossary**

**Action potential (AP):** electrical activity created by the changes in voltage across the membrane of cells. The AP of cardiac cells is composed of different phases: resting phase, depolarisation, early repolarisation, plateau phase and repolarisation. The length of time the cell remains depolarised above the resting potential is called the action potential duration (APD) and, in the event of having an abnormal APD due to a delayed repolarisation phase, this APD is said to be prolonged.

**Afterdepolarisation:** an abnormal depolarisation of cardiomyocytes that can interrupt the different phases of the cardiac AP, creating arrhythmogenic events. These can be early afterdepolarisations (EADs), where secondary voltage depolarisations occur during the repolarising phase of the AP. EADs can occur following an interruption or delayed repolarisation, which can cause lethal cardiac arrhythmias. Delayed afterdepolarisations (DADs) occur in late phase 3 or early phase 4 when the AP is nearly or fully repolarised.

**Arrhythmia:** abnormal heartbeat caused by impaired electrical conduction in the heart.

**Atrial fibrillation:** a type of arrhythmia that starts in the upper chambers of the heart (the atria), leading to an irregular and fast heartbeat.

**Ca^2+^-sensitive organic dyes:** chemical compounds (e.g. fura-4F or fluo-4 AM) that chelate free Ca^2+^, leading to either a change in fluorescence intensity or an excitation/emission wavelength shift. These dyes are cell permeable and can easily enter into hiPSC-CMs, and are regularly used to evaluate changes in Ca^2+^ flux in these cells. Some caution in their use is required because these indicators are buffers and as such can alter cellular physiology, and not all Ca^2+^-regulating components found in an adult cardiomyocyte (e.g. t-tubules) are present in hiPSC-CMs.

**Cardiac hypertrophy:** thickening of the heart muscle due to an increase in cardiomyocyte size, resulting in a decrease in size of the chambers of the heart.

**Cardiac muscle:** striated muscle tissue found in the heart. The striations are due to the presence of sarcomeres, the smallest contractile units formed by myofibrils. The precise alignment of these filaments results in a striated appearance that is observable by light microscopy as alternating light and dark bands. The dark bands are referred to as the A-band and are thick filaments of myosin, whereas the light band is known as the I-band, and is a zone of thin filaments of actin.

**Cardiac sodium channel (Na_v_1.5):** a voltage-gated transmembrane ion channel expressed in cardiac cells that controls the flow of sodium (Na^+^ ) into the cell and is responsible for the Na^+^ current (*I*_Na_). The opening on the channel is responsible for the fast upstroke of the AP and occurs during the depolarisation phase of the AP. Following depolarisation of the cell, the channel quickly closes but does not become completely inactive as a very small noninactivating current [late *I*_Na_ (*I*_NaL_)] persists during the plateau phase of the AP). Genetic mutations or drugs blocking this channel can cause both a loss or gain of function.

**Desmosomes:** also known as macula adherens: cell structures that result in cell-to-cell adhesion. They form part of the intercalated discs between cardiomyocytes, forming a mechanical link between intermediate filaments of the cell cytoskeletons of adjacent cells to stop separation during contraction.

**Electrical-field stimulation:** providing an external electrical current to the cardiomyocytes at a set frequency so that the cells relax and contract rhythmically – i.e. paced. Typically, hiPSC-CMs are paced at ∼1 Hz because this is the physiological frequency for a resting adult human heart.

**Embryoid body (EB):** three-dimensional aggregate of PSCs that can differentiate into cell types from all three germ lineages. Exposing the EBs to similar signals that occur during embryonic development, the differentiation of the EBs can be directed towards certain cell types, such as cardiomyocytes.

**END-2 cells:** visceral endodermal-like cells derived from mouse P19 embryonal carcinoma cells. When hPSCs are co-cultured with these cells, signalling molecules secreted by these cells induce hPSCs to form cardiomyocytes.

**Field-potential duration (FPD):** measured using a multielectrode array system using extracellular electrodes. With cardiac cells, the FPD corresponds to the APD, and can be correlated to the QT interval on an electrocardiogram.

**Genetically encoded calcium indicator (GECI):** a synthetic protein (e.g. GCamp6f, R-GECO1) consisting of a fluorescent protein fused with a Ca^2+^-binding protein. When Ca^2+^ binds to the GECI, a conformational change occurs, resulting in a change in fluorescence. These genetic reporters can be readily transfected into hPSC-CMs and potentially be targeted to specific cardiac subtypes.

**Genetically encoded voltage indicator (GEVI):** synthetic protein (e.g. ArcLight, ASAP1, QuasAR) that can detect changes in the membrane potential (voltage) of a cell, and reports these changes through variations in fluorescence intensity. Like voltage-sensitive organic dyes, these optogenetic reporters provide an alternative to patch clamp, but have slower response times than dyes. They do, however, offer the advantage that specific cardiac populations can be targeted and also provide a more homogenous signal.

**Heterologous cell culture systems:** *in vitro* system in which a gene is overexpressed in a cell line that does not express it. This model has been used to investigate genetic cardiac diseases by ectopically expressing mutant proteins in a non-cardiac cell (e.g. HEK cells) and assessing the resulting phenotype. However, the lack of the same cellular context as a cardiomyocyte is a disadvantage of this approach.

**Heterotypic cell model:** an *in vitro* model created by incorporation of different cell types. They can be used to establish synthetic tissues (e.g. cardiac microtissues) that more closely resemble the cellular composition of the tissue *in vivo*. Such systems will be powerful tools for studying diseases with multicellular contributions and that are not cell autonomous because it will simulate the cross-talk between the different cell types.

**Implantable cardioverter defibrillator:** a small battery-powered device that is placed subcutaneously in the chest or abdomen to monitor heart rate. If an abnormal heart rhythm is detected, the device will generate an electric shock to restore a normal heartbeat.

**L-type calcium channel:** voltage-gated transmembrane ion channel that controls the flow of Ca^2+^ ions into the cell. This current (*I*_CaL_) leads to the plateau phase of the AP and is responsible for triggering Ca^2+^ release from the sarcoplasmic reticulum.

**Left-ventricular outflow-tract obstruction:** congenital heart defect in which the ventricular outflow tract that is connected to the aorta is blocked or obstructed. If not treated, this can lead to hypertrophy and failure of the left ventricle.

**Maximal respiratory capacity:** a measurement indicating the maximum capability of the cell to respond to an energetic demand. It is calculated by adding a compound to uncouple oxygen consumption from ATP production in the mitochondria and measured using an instrument that records the level of O_2_ consumption following this stimulation in energy demand. It is often used as a parameter to assess mitochondrial dysfunction in hPSC cardiac disease models.

**Multielectrode array (MEA):** glass slides containing microscopic metal electrodes distributed on a small surface area that measure the extracellular field potential, a surrogate measure of the QT interval. Because the measurements are non-invasive, clusters of hPSC-CMs can be assayed multiple times over several weeks. The incorporation of sharp electrodes to penetrate the cell membrane also allows APs to be recorded, although poor seal formation with the cell membrane means that it currently cannot replace patch clamp electrophysiology.

**Myocardial infarction:** damage to the heart muscle due to decreased blood flow to part of the heart. It is commonly known as a heart attack.

**Patch-clamp electrophysiology:** technique that enables the AP of hiPSC-CMs to be recorded, as well as how individual ion channels behave in both healthy and disease hiPSC-CMs. An electrode forms a tight seal with the cell membrane, allowing changes in voltage and current to be measured. Such measurements can be performed manually by a trained electrophysiologist measuring the cells adhered to a substrate, or automated, where the cells are measured in suspension by an automated system.

**QT interval:** the time between the start of the Q wave and the end of the T wave in the heart's electrical cycle when measured using an electrocardiogram. The QT interval represents the electrical depolarisation and repolarisation of the ventricles. The QT interval is said to be prolonged (long QT) if it is >440 ms in men or >460 ms in women, and abnormally short (short QT) if it is <350 ms. Both conditions can have a genetic basis or can be drug induced. They can lead to irregular beating of the heart and an increased risk of sudden cardiac death.

**Syncope:** sudden loss of consciousness due to a disorder of heart rhythm. With cardiovascular syncope, the heart rate slows, causing a decrease in blood flow to the brain and leading to fainting. Serious heart conditions with an impaired electrical conduction system (e.g. LQTS) are prone to cause syncope.

**Voltage-gated potassium channels:** transmembrane ion channels sensitive to voltage changes that control the flow of potassium ions (K^+^) during the AP. The rapid and slow delayed rectifier potassium channels, which conduct the currents *I*_Kr_ and *I*_Ks_, respectively, play a crucial role in the repolarisation of the cardiomyocyte.

**Voltage-sensitive organic dyes:** chemical compounds (e.g. di-4-ANEPPS, di-4-ANBDQBS and fluovolt) that intercalate into the lipid bilayer of the plasma membrane and can be used to indicate membrane potential (voltage) changes through variations in fluorescence intensity. They are used in combination with high-speed (∼1000 fps) cameras and, because the response times of these dyes are fast, their change in fluorescence intensity profile resembles that of an AP obtained by patch-clamp electrophysiology, although absolute values are not obtainable by this approach. Also, some of these dyes result in phototoxicity and photobleaching of the cell, which limits their prolonged use.

## Functional assessment of hPSC-CMs

**Fig. 1.**
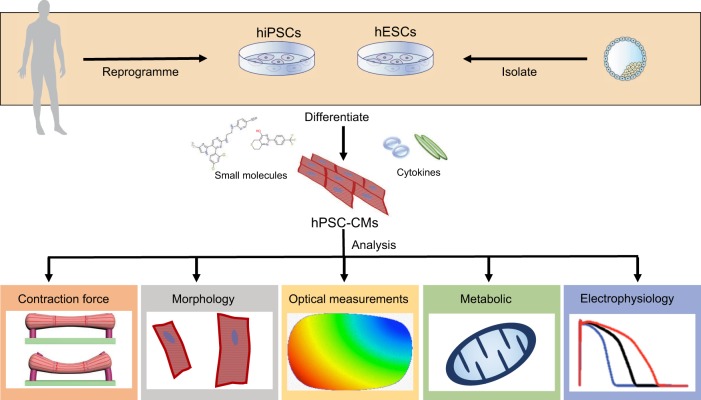
**Examples of phenotypic properties that can be quantitatively assessed in human pluripotent stem cell-derived cardiomyocytes (hPSC-CMs).** Human iPSCs, generated by reprogramming adult somatic cells, or ESCs isolated from human blastocysts, can be differentiated into cardiomyocytes using small molecules, cytokines or a combination of both. The resulting cardiomyocytes can then be used in downstream assays to measure their contractility, electrophysiology, Ca^2+^ flux, mitochondrial function or morphology. If the hPSC-CMs contain genetic mutations associated with cardiac disease, this can provide insight into the underlying disease mechanisms and also enable new therapeutic strategies to be evaluated.

A crucial requirement in the development of cell-based models of cardiac disease is the availability of reliable methods for hPSC-CM generation and for the evaluation of cardiomyocyte phenotypes in a disease context (Fig. 1). The last decade has seen a dramatic improvement in methods for generating hPSC-CMs, including those derived from patients with cardiovascular disorders (see Box 2 for an overview of differentiation strategies), and it is now possible to generate these cells in sufficient quantities and with adequate purity to use them in a wide variety of assays. Table 1 lists different techniques that have been used, and provides examples as well as key strengths and weaknesses of these procedures. Electrophysiology and analysis of ion-channel properties are among the most commonly used methods. The cardiac action potential (AP; Box 1) measured in hPSC-CMs can reflect the contribution of that particular cardiomyocyte subtype (ventricular-, atrial- or nodal-like) to the electrocardiogram (ECG) profile recorded in patients with congenital heart disorders. For example, long QT syndrome (LQTS) can lead to an extended QT interval on an ECG (Box 1) (Moss and Kass, 2005). This characteristic is also reflected in patient hiPSC-CMs as a prolongation of the AP duration (APD) because the majority of ion channels involved in generating the AP are expressed in hiPSC-CMs (Bellin and Mummery, 2016). The gold-standard approach to obtaining these measurements uses manual patch-clamp electrophysiology (Box 1). However, this approach is technically demanding, requiring a skilled operator, and has very low throughput (Denning et al., 2016). Automated patch-clamp platforms can increase throughput, enabling up to 384 cells to be measured simultaneously (Obergrussberger et al., 2016); this method is increasingly used for measuring individual currents in hPSC-CMs, although presently not APs (Rajamohan et al., 2016). A compromise between sensitivity and high-throughput is provided by the multielectrode array (MEA; Box 1) (Asakura et al., 2015). This medium-throughput method can detect disease phenotypes in LQTS hPSC-CM models, as well as drug responses.

**Box 2. Generation of hPSC-CMs**

Initial strategies for differentiating hPSCs to cardiomyocytes relied on either co-culture with endodermal stromal cells (END-2) or by embryoid body (EB) differentiation (Box 1) (Mummery et al., 2012). However, the yield of cardiomyocytes was low, and protocols were time consuming and poorly reproducible owing to heterogeneity in the size of hPSC clusters in culture and the inclusion of undefined components such as serum (van den Berg et al., 2014). Later protocols therefore focussed on generating cardiomyocytes from EBs containing defined numbers of cells, in serum-free medium with timed exposure to cytokines known to play a role in embryonic heart development (Burridge et al., 2011; Elliott et al., 2011; Kattman et al., 2011; Yang et al., 2008). Although these methods have improved reproducibility and efficiency, the yield of cardiomyocytes relative to the starting number of hPSCs is still low. To simplify differentiation procedures, strategies have subsequently been developed to take advantage of the two-dimensional monolayer format that undifferentiated hPSCs are maintained as. The same signalling cues are required in monolayer differentiation as in EB-based procedures (Kadari et al., 2015; Lian et al., 2012; Paige et al., 2010; van den Berg et al., 2014) but, because in this format the cells are more uniformly exposed to the differentiation signals, higher percentages and yields of cardiomyocytes can be obtained (Zhang et al., 2012). Furthermore, the growth factor supplements in monolayer differentiations can be completely replaced with small molecules that exhibit less lot-to-lot variation compared to cytokines (Burridge et al., 2014; Gonzalez et al., 2011; Kim et al., 2015; Lian et al., 2015; Minami et al., 2012). These small-molecule protocols have also been adapted to hPSCs cultured in suspension (Kempf et al., 2014; Kempf et al., 2015). This will facilitate the upscaling of hPSC-CM differentiation in larger volumes, and potentially at a capacity necessary for therapeutic and industrial applications. Overall, the improvements in differentiation techniques, as well as their reproducibility across multiple cell lines, has almost entirely eliminated issues associated with generating cardiomyocytes from hPSCs in sufficient numbers.

**Table 1.**
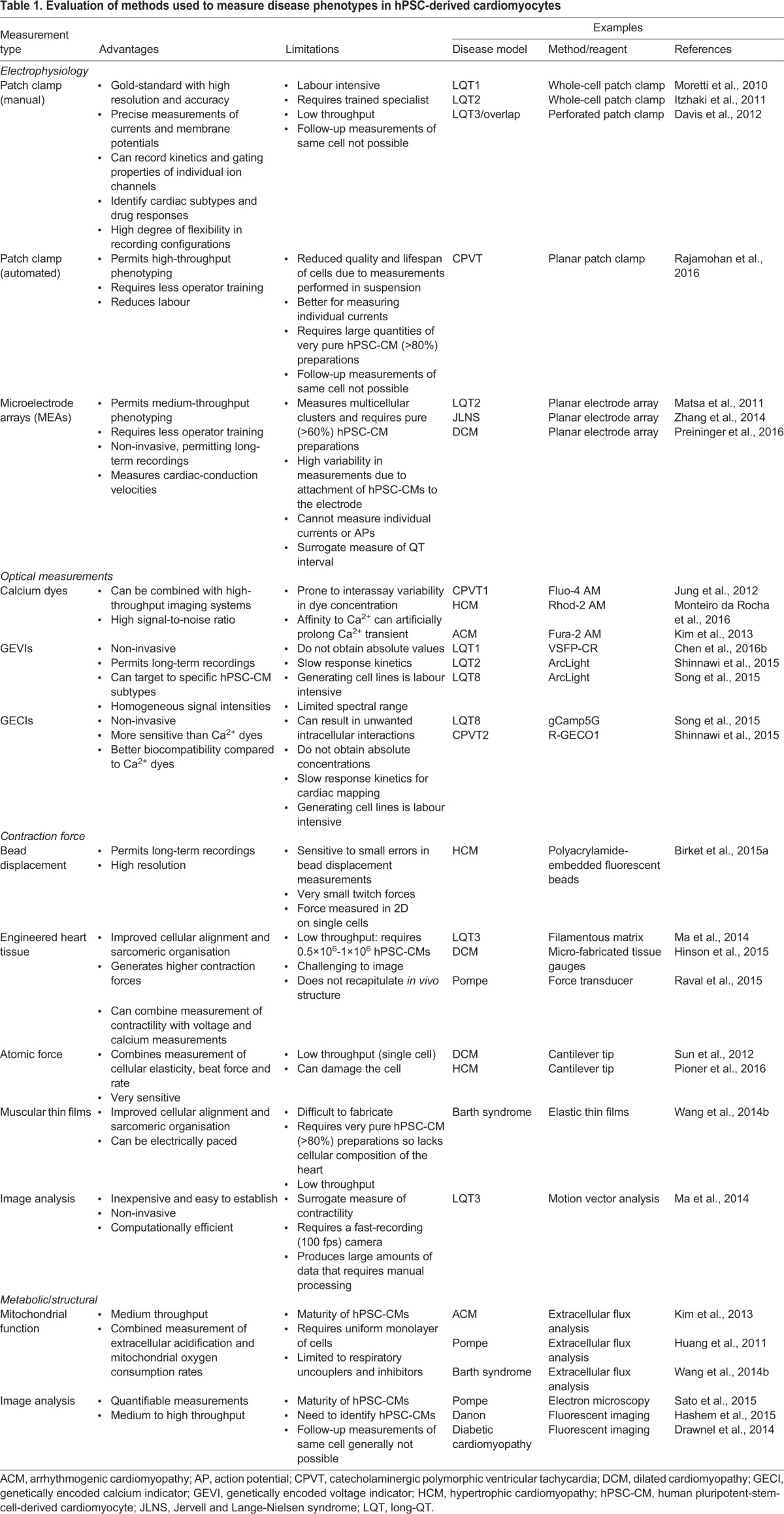
**Evaluation** **of methods used to measure disease phenotypes in hPSC-derived cardiomyocytes**

Optical-based approaches using fluorescence-based voltage- or calcium-sensitive indicators (organic dyes) provide an alternative method for measuring changes in membrane potential and calcium flux in hPSC-CM disease models (Box 1). Unlike patch clamp, voltage-sensitive organic dyes do not provide absolute values. These dyes can also be combined with Ca^2+^ indicators, permitting simultaneous imaging of electrical and calcium dynamics (Lee et al., 2012). Because Ca^2+^ cycling in the cardiomyocytes converts electrical excitation into force generation, Ca^2+^ probes are frequently used to detect Ca^2+^ dysfunction in hPSC-CM disease models, such as those modelling cardiac hypertrophy (Box 1). Nevertheless, both Ca^2+^- and voltage-sensitive indicators suffer from some collective limitations, including interassay loading variability and lack of suitability to target specific cardiomyocyte subtypes (van Meer et al., 2016). With recent advances in the development of genetically encoded voltage or calcium indicators (GEVIs or GECIs, respectively; Box 1) (Lin and Schnitzer, 2016), this suggests that these could be an effective alternative for functionally assessing hPSC-CMs, and several have been used to detect APs and Ca^2+^ transients in hPSC-CMs, including in disease models (Table 1).

There are also numerous techniques to measure contractility – which is disturbed in many cardiac diseases – in hPSC-CMs. Because each approach quantifies force differently, cross-comparison of measurements is not possible. Measurements are performed either on individual hPSC-CMs or on two- or three-dimensional cell clusters, and have been used to assess contractile dysfunction in hPSC models of cardiomyopathies (Table 1). Not surprisingly, out of these models, three-dimensional engineered heart constructs mimic native cardiac tissue best (Eschenhagen et al., 2015). Indeed, reduced contractility in an hiPSC line derived from a cardiomyopathy patient with a mutation in the sarcomeric protein titin could only be detected when the cardiomyocytes were cultured in three-dimensional aggregates (Hinson et al., 2015). However, the forces generated by hPSC-CMs even in these multicellular constructs remain much smaller than those of adult cardiomyocytes and exhibit different degrees of contractile deficits compared to human hearts with titin mutations (Hinson et al., 2015; Hirt et al., 2014a). An additional limitation of many of these contractility assays is their low throughput (van Meer et al., 2016).

High-spatial and -temporal resolution microscopy has also become an essential tool for analysing cardiac disease phenotypes because it allows cardiomyocyte analysis down to the organelle level (Denning et al., 2016) (Table 1). Imaging can be used to evaluate changes in cardiomyocyte morphology, such as hypertrophic phenotypes (Kriston-Vizi et al., 2017). These systems can also be used to assess other phenotypes such as mitochondrial content and organization, which are affected in certain cardiomyopathies (Lee et al., 2016; Lin et al., 2015). Mitochondrial (dys)function in hPSC-CM disease models can also be evaluated by measuring glycolysis and oxidative phosphorylation (Denning et al., 2016) (Table 1).

These recent technological advances in both generating and evaluating hiPSC-CMs have already had a major impact on our understanding of cardiac diseases, as described in the following section.

## hPSC models of cardiac diseases

**Fig. 2.**
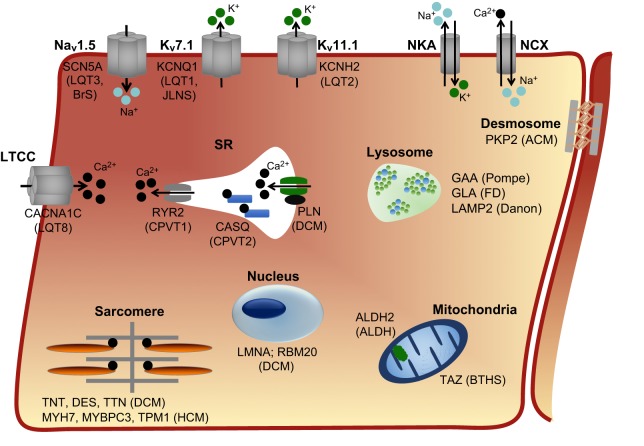
**Overview of congenital cardiac diseases that have been modelled using hPSC-CMs.** The main cellular sublocalisation of the protein affected in each disease is indicated. The diseases caused by defects in each protein are shown in brackets. A more extensive list of the mutations that have been examined is provided in Table 2. ACM, arrhythmogenic cardiomyopathy; ALDH, aldehyde dehydrogenase; BrS, Brugada syndrome; BTHS, Barth syndrome; LTCC, L-type calcium channel; CPVT, catecholaminergic polymorphic ventricular tachycardia; DCM, dilated cardiomyopathy; FD, Fabry disease; HCM, hypertrophic cardiomyopathy; JLNS, Jervell and Lange-Nielsen syndrome; LQT, long QT syndrome; K_v_7.1, voltage-gated, slow rectifier potassium channel; K_v_11.1, voltage-gated, fast rectifier potassium channel; Na_v_1.5, voltage-gated cardiac sodium channel; NCX, sodium/calcium exchanger; NKA, sodium/potassium exchanger; SR, sarcoplasmic reticulum.

Despite improved understanding of the genetics underlying cardiac diseases, treatment options (e.g. drug therapies) are still limited or else act primarily to delay disease progression (Chandrasekera and Pippin, 2015). Furthermore, tailoring treatments to patients based on their genetic mutation and risk – a key goal of precision medicine – is yet to become a reality. However, hPSC-CMs are now being used to model a wide range of cardiac disorders (Fig. 2), not only to investigate the underlying disease mechanisms but also to evaluate therapeutic options in a patient-specific manner. Although arrhythmias and cardiomyopathies continue to be the most commonly investigated cardiac diseases, cardiometabolic disorders and more complex conditions without clear genetic causes are also being modelled. Table 2 summarises the published hPSC models of these three groups of disorders as well as the strategies used to ameliorate the disease phenotype and their applicability for treating patients. Below, we review some of the key novel findings from hPSC-CM disease models, both from a mechanistic and clinical perspective. For a discussion of hPSC models of non-cardiovascular-specific diseases with cardiac traits, we refer the reader to the following reviews (Chen et al., 2016a; Giacomelli et al., 2017).

**Table 2.**
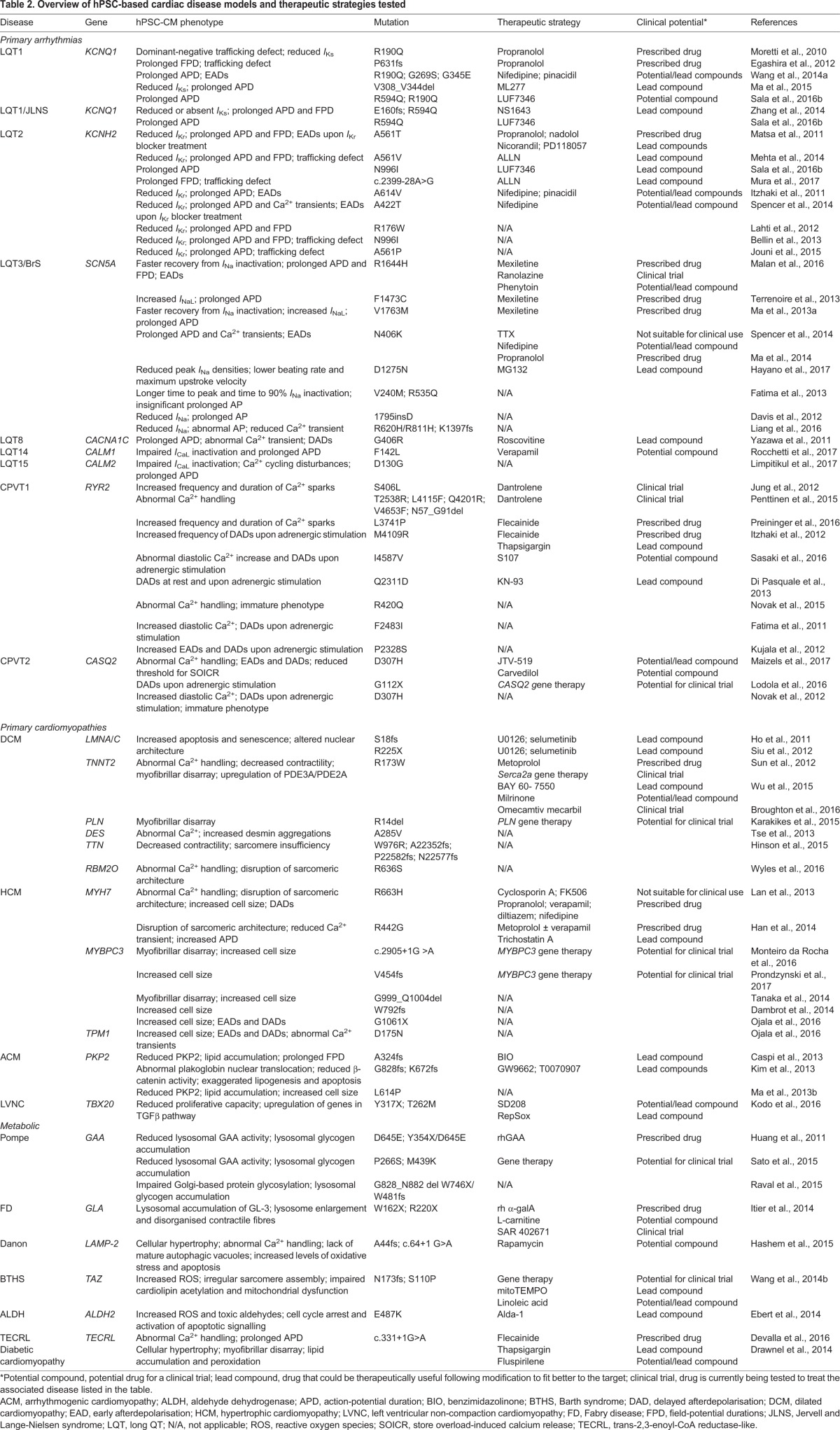
**Overview of hPSC-based cardiac disease models and therapeutic strategies tested**

## Primary arrhythmic diseases

Inherited channelopathies caused by mutations in cardiac ion channels are a group of diseases that have been extensively modelled using hPSC-CMs. These arrhythmic disorders include LQTS, Jervell and Lange-Nielsen syndrome (JLNS), Brugada syndrome (BrS), and catecholaminergic polymorphic ventricular tachycardia (CPVT). The hallmark feature of channelopathies is an abnormal ECG, either at baseline (without a trigger) or, for example, during exercise (Marsman et al., 2013). This can predispose patients to an increased risk of cardiac arrhythmias, syncope (Box 1) and even sudden cardiac death (SCD).

### LQT1

One of the first cardiac-disease hiPSC models was generated from LQT1 (a type of LQTS) patients with a missense mutation (R190Q) in *KCNQ1*, which encodes a voltage-gated potassium ion channel (Box 1) (Moretti et al., 2010)*.* The hiPSC-CMs showed a 70-80% reduction in the slow component of the delayed rectifier potassium current (*I*_K__s_) compared to cells obtained from healthy controls, a corresponding prolongation in the APD, and development of early afterdepolarisation (EAD; Box 1) events in the presence of the β-adrenergic agonist isoproterenol, which is an arrhythmic trigger in LQT1 patients. These phenotypes reflect electrophysiological features of the disorder observed in patients (Giudicessi and Ackerman, 2012). Another frameshift mutation in *KCNQ1* was later shown to cause a similar electrophysiological phenotype and response to adrenergic stimulation in patient hiPSC-CMs (Egashira et al., 2012). In both cases, EADs were blunted in hiPSC-CMs by pretreatment with the β-blocker propranolol. This correlated well with clinical observations where β-blocker treatment is the first line of therapy in suppressing arrhythmias in LQT1 patients (Ruan et al., 2008), and indicated that hiPSC-CMs may be valuable in developing novel treatments for this disease.

Demonstrating this, ML277, a compound identified as a potent activator of KCNQ1 channels (Mattmann et al., 2012), was shown to partially shorten APDs in hiPSC-CMs from LQT1 patients and healthy individuals (Ma et al., 2015). However, it is important to note that KCNQ1 forms channel complexes with β-subunits of another potassium channel, KCNE1, and it is unclear whether the stoichiometry of this is the same in both immature hiPSC-CMs and adult hearts (Yu et al., 2013). Because this could affect the efficacy of ML277, validating the compound in more mature wild-type and LQT1 hiPSC-CMs will assist in determining whether it could become a targeted drug for LQT1. Similarly, a recent study investigated whether a novel allosteric modulator (LUF7346) of the voltage-gated K^+^ channel, hERG, could be used to treat congenital and/or drug-induced forms of LQTS (Sala et al., 2016b). LUF7346 acts as a type-1 hERG activator by increasing the rapidly activating delayed rectifier K^+^ current (*I*_Kr_) window and slowing *I*_Kr_ deactivation in a voltage-dependent manner (Yu et al., 2015). By genetically correcting hiPSCs that harbour the KCNQ1 mutation R190Q, a pair of isogenic lines (LQT1^corr^/LQT1^R190Q^) was created, thereby eliminating the influence of genetic background on the drug response (Sala et al., 2016b). Treatment with 3-5 µM LUF7346 significantly shortened the APD in the LQT1 hiPSC-CMs, highlighting the potential of hERG allosteric modulation for treating congenital LQTS. Higher doses, however, stopped spontaneous beating and increased the risk of excessive QT-interval shortening. Further investigation is needed to determine whether this will present an obstacle for clinical translation.

### JLNS

JLNS is also caused by mutations in *KCNQ1*, although it is a recessive disorder whereas LQT1 is an autosomal dominant or sporadic disease. JLNS patients display particularly severe cardiac symptoms and cannot be sufficiently protected by β-blocker therapy (Schwartz et al., 2006). By combining patient-derived hiPSCs with genetic engineering, a collection of heterozygous and homozygous hiPSCs for two different classes of JLNS-causing mutations (a missense and a putative splice-donor mutation) was generated (Zhang et al., 2014). Cardiomyocytes from both JLNS hiPSC lines showed similar repolarisation defects, despite the molecular consequence of each mutation being different. Treatment with another hERG activator, NS1643, shortened field-potential durations (FPDs; Box 1) and protected both JLNS hiPSC lines from drug-induced arrhythmias (Zhang et al., 2014). Comparable reductions in FPD were also observed with LUF7346 (Sala et al., 2016b), suggesting that a single drug might be able to protect against multiple forms of *KCNQ1*-linked disorders. However, both NS1643 and LUF7346 have steep dose-response curves, so it may be difficult to find a dose that does not cause short QT.

### LQT2

Another LQTS subtype, LQT2, is caused by mutations in the potassium-channel gene *KCNH2* that lead to a reduction in *I*_Kr_. Similarly to LQT1, LQT2 hiPSC-CMs exhibit prolonged APD, arrhythmogenic events and irregular beating, thus reflecting typical aspects of the patient phenotype (Itzhaki et al., 2011; Matsa et al., 2011). Moreover, as in LQT1, treatment of LQT2 hiPSC-CMs with β-blockers can correct EADs caused by adrenergic stimulation or pharmacological blockade of cardiac repolarisation currents (e.g. E4031) (Matsa et al., 2011). However, not all β-blockers are equally as effective at preventing breakout cardiac events in LQTS patients (Wilde and Behr, 2013). Being able to test alternative drugs on a range of LQT2 hPSC lines could improve treatment strategies and also create opportunities to develop tailored therapies for patients depending on their mutation and genetic background. For example, Itzhaki et al. demonstrated that the clinically approved compounds nifedipine (a Ca^2+^-channel blocker) and pinacidil (a K_ATP_-channel activator) shortened the APD and FPD and abolished EADs in hiPSC-CMs from an LQT2 patient (Itzhaki et al., 2011). However, both compounds have a risk of causing hypotension, which could limit their clinical applicability for this disease (Kamp, 2011).

Treating LQT2 patients with molecules that activate *I*_Kr_ is also of particular interest and several compounds have been identified that have a similar effect on *I*_Kr_ but act through different mechanisms (Yu et al., 2015). Matsa et al. examined the response of hiPSC-CMs to two experimental K^+^-channel enhancers: nicorandil and PD-118057 (Matsa et al., 2011). Both drugs shortened the prolonged APD of LQT2 hiPSC-CMs. However, as with LQT1, dosage needs to be carefully monitored to avoid excessive shortening of the AP. More novel treatments have also been proposed, such as the chaperone modulator *N*-[*N*-(*N*-acetyl-L-leucyl)-L-leucyl]-L-norleucine (ALLN). This small molecule leads to re-trafficking of hERG and rescue of the LQT2 phenotype in an hiPSC model (Mehta et al., 2014). mRNA knockdown by mutated-allele-specific RNA interference was also shown to rescue the disease phenotype (Matsa et al., 2014). Although neither of these approaches is directly translatable to the clinic, these findings underline the importance of understanding the complexity of different genetic defects at the molecular and cellular levels to develop alternative treatment strategies.

### LQT3 and BrS

LQT3, another subtype of LQTS, is caused by gain-of-function mutations in *SNC5A*, which encodes the α-subunit of the cardiac sodium channel, Na_v_1.5 (Box 1). These mutations lead to the sodium current (*I*_Na_) failing to inactivate properly, thereby increasing APD and prolonging cardiomyocyte repolarisation (Ruan et al., 2014). β-blocker therapy in LQT3 patients is less effective than in other LQTS types, and in some instances can be harmful owing to other underlying disorders (Remme et al., 2008). This is because *SCN5A* mutations are also associated with loss-of-function arrhythmic disorders, including BrS and conduction disease (Remme et al. 2008). These loss-of-function diseases are due to a decreased peak *I*_Na_, which leads to slower AP upstrokes. Some *SCN5A* mutations even result in the combination of several clinical manifestations and are commonly referred to as ‘overlap syndromes’ (Remme et al., 2008). However, associating different *SCN5A* mutations with particular phenotypes has been challenging owing to difficulties in accurately modelling some of these mutations using heterologous cell culture systems (Box 1) (Davis et al., 2012; Mohler et al., 2004). We demonstrated the potential of hiPSC-CMs as an alternative model by establishing that, despite their immaturity, these cells displayed features of both BrS and LQT3 (Davis et al., 2012). More recently, Liang et al. (2016) showed that hiPSC-CMs can model *SCN5A* mutations that cause only BrS and, by genome editing, they were able to correct one variant and validate its pathogenicity.

Terrenoire et al. (2013) further demonstrated the possibility to use hiPSCs to develop personalised treatment regimens using an hiPSC line derived from an LQT3 patient with a *de novo* mutation (F1473C) in *SCN5A* and a polymorphism (K891T) in *KCNH2*. An implantable cardioverter defibrillator (ICD; Box 1) and high doses of the Na^+^-channel blocker mexiletine and propranolol helped reduce the numbers of arrhythmias experienced by the patient; however, multiple episodes were still detected daily. The authors first used hiPSC-CMs to demonstrate that the disease was primarily due to the *SCN5A* mutation and not the *KCNH2* polymorphism. Treating the hiPSC-CMs with high doses of mexiletine led to both an anti-arrhythmic drug block of *I*_NaL_ plus a pro-arrhythmic block of *I*_Kr_, providing an explanation for the recurrent cardiac episodes observed in the patient. Although Na^+^-channel blockers can be beneficial in treating LQT3, this depends on how the mutation affects the biophysical properties of Na_v_1.5. Indeed, testing these channel blockers in hiPSC models of different *SCN5A* mutations has highlighted their differing degrees of effectiveness (Ma et al., 2013a; Malan et al., 2016), though the genetic background of the cell lines might also influence this.

### LQT8

LQT8, also known as Timothy syndrome, is a very rare, multisystem LQTS subtype caused by a single-amino-acid substitution in exon 8a of *CACNA1C*, which encodes a subunit of the L-type Ca^2+^ channel (Box 1) (Splawski et al., 2004). At the cellular level, the mutation causes impaired inactivation of the channel, resulting in a persistent inward current that prolongs the APD (Yazawa et al., 2011). Although treatment with the Ca^2+^-channel blocker verapamil, β-­blockers or the Na^+^-channel blocker ranolazine show some beneficial effects, the majority of LQT8 patients die before puberty from cardiac arrhythmias (Venetucci et al., 2012). Ca^2+^ imaging of LQT8 hiPSC-CMs revealed excess Ca^2+^ influx and abnormal Ca^2+^ transients (Yazawa et al., 2011). Additionally, irregular contraction, prolonged APD and an increased incidence of delayed afterdepolarisations (DADs) were recorded. Roscovitine, a cyclin-­dependent kinase inhibitor, was able to correct most of the alterations caused by channel dysfunction, validating earlier cellular studies (Yarotskyy et al., 2009). However, owing to its inhibition of multiple proteins involved in the cell cycle (Meijer et al., 1997), roscovitine is more likely to serve as a lead compound for developing new antiarrhythmics rather than as a potential treatment for LQT8 patients.

### CPVT

CPVT is an arrhythmogenic disorder that is also characterised by abnormal intracellular Ca^2+^ handling and signalling in cardiomyocytes. It causes DADs through the activation of the membrane Na^+^/Ca^2+^ exchanger (NCX) (Wehrens, 2007). Clinically, CPVT is triggered by situations that increase the level of catecholamines (e.g. norepinephrine), such as physical exertion and emotional stress. CPVT1 is the most common type of CPVT and is caused by autosomal dominant mutations in the cardiac ryanodine receptor type 2 gene (*RYR2*), a mediator of calcium release in the sarcoplasmic reticulum (SR) (Priori and Chen, 2011). CPVT2 is a rarer, autosomal recessive form caused by mutations in the calsequestrin-2 gene (*CASQ2*), which encodes a calcium-binding protein also located in the SR (Lahat et al., 2001). Treatment of CPVT generally consists of β-blocker therapy, although 30% of patients still experience life-threatening arrhythmias (Cerrone et al., 2012). It is therefore important to understand the functional consequences of a particular mutation to develop individualised treatments, particularly because both CPVT1 and CPVT2 have a similar clinical presentation yet different disease mechanisms.

CPVT hiPSC-CMs exhibited similar phenotypes to those observed in the patients, with all mutations appearing to cause aberrant Ca^2+^ transients and the development of DADs, which in some cases were exacerbated with adrenergic stimulation (Di Pasquale et al., 2013; Itzhaki et al., 2012; Jung et al., 2012; Kujala et al., 2012; Novak et al., 2015; Preininger et al., 2016). As observed clinically, the Na^+^­ channel blocker flecainide restored intracellular ion concentration to normal levels in the hiPSC-CMs (Itzhaki et al., 2012). These models have also provided insight into the disease mechanism. It is proposed that *RYR2* mutations render the ryanodine receptors ‘leaky’ following protein kinase A (PKA)-mediated phosphorylation, producing local depolarisations that trigger DADs via activation of NCX (Wehrens et al., 2003). An alternative theory is that *RYR2* mutations can result in SR Ca^2+^ overload following β-adrenergic exposure, resulting in abnormal release of Ca^2+^ independent of FK506-binding protein (FKBP) modulation and leading to a similar electrophysiological phenotype (Jiang et al., 2005). Both of these mechanisms have been reported in hiPSC CPVT1 models (Itzhaki et al., 2012; Zhang et al., 2013), suggesting that the position of the mutation in *RYR2* plays a key role in the underlying cause of the abnormal Ca^2+^ handling and the different drug responses observed in patients. For example, dantrolene, a drug used to treat malignant hyperthermia, abolished or reduced arrhythmias in patients where the *RYR2* mutation was in the N-terminal or central region, whereas no effect was seen when the mutation was in the transmembrane region (Penttinen et al., 2015). These responses were also observed in hiPSC-CMs generated from each of these patients. Several other novel treatments of CPVT1 have also been reported, including thapsigargin [a sarco/endoplasmic reticulum Ca^2+^-ATPase (SERCA) inhibitor] and KN-93 [an antiarrhythmic drug that inhibits Ca^2+^/calmodulin-dependent protein kinase II (CaMKII)], which can both rescue the arrhythmic phenotype induced by catecholaminergic stress (Di Pasquale et al., 2013; Itzhaki et al., 2012). Neither of these compounds is likely to be clinically suitable owing to their lack of target and tissue-specificity, but they are potentially useful lead compounds.

## Cardiomyopathies

Inherited cardiomyopathies are a second group of cardiac disorders that have been widely studied using hiPSCs. Mutations in more than 50 genes have been linked to dilated (DCM), hypertrophic (HCM) and arrhythmogenic (ACM) cardiomyopathies (Wilde and Behr, 2013). Most of these disorders are characterised by sarcomeric disorganisation, which can lead to reduced myocardial function and potentially heart failure. These diseases are also marked by large variability in clinical phenotype, with some patients remaining asymptomatic throughout their lifetime, to SCD occurring in others during adolescence. Currently, treatments are typically initiated once the patient becomes symptomatic. Understanding the pathological mechanisms underlying these diseases, and in particular the remodelling of the heart that often occurs before clinical symptoms are apparent, will help in the development of earlier treatments to prevent disease progression. In this regard, it is anticipated that hiPSC-CM cardiomyopathy models will prove very useful.

### DCM

DCM is one of the most common cardiomyopathy subtypes; familial DCM has an estimated prevalence between 1 in 250 and 1 in 2500 individuals (Hershberger et al., 2013). The disease is clinically characterised by ventricular dilation and impaired contraction. More than 30 genes involved in various genetic pathways, including sarcomere and cytoskeleton formation and contraction, nuclear envelope stability, gene processing and transcription, and calcium handling, have been identified in DCM (Hershberger et al., 2013). DCM inheritance is usually autosomal dominant, with mutations in titin (*TTN*) being most frequently identified (Ingles and Semsarian, 2014). Patients with familial DCM are treated with angiotensin-converting enzyme (ACE) inhibitors, β-blockers and diuretics similar to those used for other systolic heart failure conditions (Leviner et al., 2015). There are currently no etiology-specific cardioprotective treatments for asymptomatic familial DCM patients.

To date, mutations in six genes have been studied using DCM-hiPSC models (Table 2). A heterozygous missense mutation (R173W) in the sarcomeric protein troponin T (TNNT2) was intensively studied with hiPSCs generated from seven family members (Sun et al., 2012). Key features of the disease were observed in the mutated hiPSC-CMs, including impaired Ca^2+^ handling, reduced contractility and downregulation of SERCA2a. Metoprolol (a β-adrenergic blocker used to treat DCM patients) decreased the number of cardiomyocytes with abnormal sarcomeric α-actinin staining, whereas transgenic overexpression of *SERCA2a*, a gene-therapy treatment for heart failure that is now in clinical trials (Greenberg et al., 2014), improved their contractile function. A follow-up mechanistic study indicated that the R173W mutation increases nuclear translocation of TNNT2 and enhances epigenetic activation of the phosphodiesterase genes *PDE2A* and *PDE3A* (Wu et al., 2015). This upregulation led to compromised β-adrenergic regulation in DCM hiPSC-CMs, resulting in contractile dysfunction. Treatment with the PDE2 and PDE3 pharmacological inhibitors BAY 60-7550 and milrinone improved calcium handling and the contractile force in DCM hiPSC-CMs. Although milrinone has been prescribed to heart failure patients for many years, recent studies have questioned its safety and efficacy (McMurray et al., 2012; Yancy et al., 2013). It will be interesting to see whether BAY 60-7550 or related PDE2 inhibitors are a better option, although currently there are no FDA-approved PDE2 inhibitors. Myofibrillar architecture was also found to be affected in the hiPSC-CMs derived from one severely afflicted family member (Broughton et al., 2016). Whether the observed sarcomeric shortening and slow actin assembly dynamics is due to the TNNT2 mutation or the presence of other genetic variants warrants further investigation. Omecamtiv mecarbil, a myosin activator previously reported to improve cardiac function in acquired heart failure (Cleland et al., 2011), reversed the phenotype by increasing contractility and improving sarcomere assembly (Broughton et al., 2016). Currently, only transplantation satisfactorily addresses depressed contractility in familial DCM. The possibility that omecamtiv mecarbil could treat this without adversely altering Ca^2+^ flux is an exciting prospect.

Other DCM hiPSC models have examined variants in the genes encoding lamin A/C (*LMNA*) and *TTN* (Table 2). LMNA-related DCM is characterised by early onset of atrial fibrillation (Box 1) and conduction disease, leading to SCD and heart failure (Fatkin et al., 1999; Pan et al., 2009). Analysis of CMs from two different LMNA variant hiPSC lines by electrical-field stimulation (Box 1) revealed increased nuclear senescence and cellular apoptosis compared to control hiPSC-CMs, potentially explaining the development of premature cardiac ageing seen in patients cardiac aging seen in patients (Ho et al., 2011; Siu et al., 2012). Pharmacological blocking of the ERK1/2 pathway with U0126 and selumetinib considerably reduced the proapoptotic effects of electric field stimulation in the mutated lines, supporting earlier animal studies that implicated this pathway as a potential therapeutic target (Muchir et al., 2012).

hiPSC-CMs have also been used to investigate the pathogenicity of different TTN-truncating variants (TTNtvs). Using hiPSC-­derived three-dimensional (3D) cardiac microtissues, Hinson et al. (2015) found that truncating mutations located within the A band of cardiac muscle (Box 1) caused more contractile deficits compared to I-band TTNtvs due to alternative exon splicing mitigating their pathogenicity. This could explain why, clinically, individuals with A-band TTNtvs are more likely to exhibit a pathogenic phenotype (Akinrinade et al., 2015), and illustrates the potential of hiPSC-CM models in prognostic evaluation. However, for hPSC-CMs to become more reliable in predicting TTNtv pathogenicity, further culture improvements are required to generate CMs that produce contractile forces more similar to those measured in adult cardiomyocytes.

### HCM

HCM is also a common cardiomyopathy subtype, with a prevalence of ∼1 in 500 individuals. It is the most common cause of SCD in young people and athletes (Leviner et al., 2015), and is clinically characterised by a thickened (≥15 mm) left ventricle, which can lead to heart failure due to diastolic dysfunction, left-ventricular outflow-tract obstruction (Box 1) or atrial fibrillation. Mutations in 23 genes encoding components of the sarcomere or sarcomeric-associated proteins have been linked to HCM, with the majority of mutations identified in β-myosin heavy chain (MYH7) and myosin-binding protein C (MYBPC3) (Frey et al., 2011). However, mutations have only been identified in ∼50% of cases, indicating that additional genes are likely to be involved. Moreover, phenotypic heterogeneity adds to the genetic complexity. Pharmacological treatment with β-blockers or verapamil can help manage the disease, but does not reverse disease progression (Frey et al., 2011).

Despite their immaturity, hiPSC-CMs derived from HCM patients with mutations in MYH7 and MYBPC3 could reproduce, in part, many characteristics of the disease, such as cellular enlargement, sarcomere disorganisation and disrupted contractility, as well as altered gene expression and calcium handling (Han et al., 2014; Lan et al., 2013). Using hiPSC-CMs with a missense mutation in *MYH7* (R663H) to screen drugs currently used to treat HCM, Lan et al. (2013) confirmed that pharmaceutical inhibition of calcium entry with verapamil prevented the development of HCM. This supports the hypothesis that dysregulation of Ca^2+^ cycling is a central pathogenic mechanism for the disease (Frey et al., 2011). A second study modelling a different missense mutation (R442G) observed similar phenotypes in the diseased hiPSC-CMs; again, improvements were seen with verapamil treatment (Han et al., 2014). Whole-transcriptome sequencing indicated that genes implicated in cell proliferation and Notch and FGF signalling were involved in disease development, highlighting potential therapeutic targets. Furthermore, the histone-deacetylase inhibitor Trichostatin A significantly ameliorated various hypertrophic phenotypes in HCM hiPSC-CMs, in line with previous animal and cellular studies (Han et al., 2014).

The majority of *MYBPC3* mutations result in a truncated, unstable protein, suggesting that the ensuing HCM phenotype is caused by haploinsufficiency (Harris et al., 2011). Using adenoviral gene transfer, it was demonstrated that expression of wild-type *MYBPC3* in an hESC line carrying a splice-donor mutation in *MYBPC3* during early cardiomyocyte differentiation prevented HCM structural and functional phenotypes (Monteiro da Rocha et al., 2016). This is similar to observations in HCM mutant mice (Mearini et al., 2014), and suggests that gene therapy could be used to treat cardiomyopathies.

### ACM

ACM is a primary cardiomyopathy characterised by ventricular arrhythmias and right-ventricle dysfunction due to fibrofatty infiltration of cardiomyocytes. It has an estimated prevalence of 1 in 5000, and, like other cardiomyopathies, displays highly variable penetrance and severity. The majority of mutations have been identified in genes encoding components of the desmosome (Box 1), most commonly in plakophilin-2 (*PKP2*) (Corrado et al., 2017). Exactly how desmosomal protein mutations lead to the ACM phenotype is unclear, although alterations to Wnt–β-catenin signalling due to impaired desmosomal assembly are thought to induce a gene transcriptional switch from myogenesis to adipogenesis and fibrogenesis (Garcia-Gras et al., 2006). Modelling ACM using hPSCs could help in further elucidating the disease pathophysiology (Sommariva et al., 2017), although the late onset of the disease and suspected involvement of epicardial cells in mediating the fibrofatty myocardial phenotype poses a challenge. By inducing adult-like metabolism in hiPSC models of *PKP2* mutations, the resulting cardiomyocytes not only displayed abnormalities in desmosome structure and gene expression, but also calcium-handling deficits and increased lipogenesis and apoptosis (Caspi et al., 2013; Kim et al., 2013). Furthermore, lipid build up was the underlying cause of pathogenesis of ACM and was due to abnormal peroxisome proliferator-activated receptor gamma (PPAR-γ) activation. This accumulation could be prevented by treating the diseased iPSC-CMs with either a GSK3β inhibitor or PPAR-γ antagonists (Caspi et al., 2013; Kim et al., 2013). The beneficial effect of inhibiting GSK3β has been observed in multiple model systems (Chelko et al., 2016), further supporting research into the therapeutic potential of this strategy.

## Metabolic disorders

Metabolic diseases are generally categorised as either inborn errors of metabolism (IEM) (i.e. inherited) or as acquired metabolic syndromes (owing to their development in adulthood from the presence of additional risk factors). With both groups, the disease typically affects multiple organs, including the heart. The cardiac complications often present as either DCM, HCM or arrhythmias, and are frequently associated with IEM disorders that affect glycogen or lysosomal storage, fatty-acid oxidation and mitochondrial metabolism or function. Distinguishing IEM as the underlying cause of the disease rather than a primary cardiomyopathy is crucial for developing disease management strategies. Therefore, hPSCs not only offer the opportunity to develop new therapeutic approaches for these diseases, but can also be used to understand how IEMs lead to cardiomyopathies. Similarly, the rise in cardiovascular disease through acquired metabolic syndromes also warrants the development of new models to better investigate these polygenic diseases (Chanana et al., 2016).

### Mitochondrial disorders

Barth syndrome (BTHS) is a mitochondrial disorder caused by mutations in the gene encoding tafazzin (*TAZ*), which acetylates the mitochondrial phospholipid cardiolipin. Impaired cardiolipin acetylation results in impaired ATP production and mitochondrial dysfunction, with one clinical consequence being cardiomyopathy (Schlame and Ren, 2006). BTHS hiPSC-CMs were derived from two patients harbouring either a missense or frameshift *TAZ* mutation (Wang et al., 2014b). Additionally, introducing *TAZ* mutations into control hiPSCs via genome editing generated an isogenic pair of cell lines. Overall, BTHS hiPSC-CMs exhibited impaired cardiolipin acetylation and mitochondrial dysfunction. The phenotypes could be reversed by gene replacement therapy whereby BTHS hiPSC-CMs were transfected with modified *TAZ* mRNA; however, maximal respiratory capacity (Box 1) was not completely rescued. Because myofilament disarray is a feature of BTHS, the authors examined sarcomeric organisation. They observed sarcomeres that were intermittent and sparse only in the BTHS hiPSC-CMs with the frameshift mutation, and not missense mutation. However, this difference, as well as variation in contractile dysfunction, could also be due to genetic background because the patients were unrelated. A dramatic improvement in sarcomeric organisation and contractile dysfunction was observed when the BTHS hiPSC-CMs were treated with the antioxidant mitoTEMPO or linoleic acid, an essential unsaturated fatty-acid precursor of mature cardiolipin (Wang et al., 2014b). Whether these small-molecule treatments can be easily translated into patient therapies remains to be seen.

Mitochondrial aldehyde dehydrogenase 2 (ALDH2) deficiency is present in about 8% of the human population, predominantly in people of East Asian heritage (Brooks et al., 2009). The ALDH2*2 polymorphism (E487K) reduces ALDH2 enzyme activity, leading to a loss of its cardioprotective effects and increasing susceptibility for coronary artery and ischemic heart disease (Guo et al., 2010). hiPSC-CMs from a cohort of East Asian individuals carrying the ALDH2*2 polymorphism demonstrated the expected accumulation of reactive oxygen species and 4-hydroxynonenal (4HNE), a toxic aldehyde product, leading to cell-cycle arrest and apoptosis signalling. Treating the ALDH2*2 hiPSC-CMs with Alda-1, a small molecule known to restore the enzymatic activity of the E487K mutant (Chen et al., 2008), rescued the apoptotic phenotype in the hiPSC-CMs (Ebert et al., 2014). Although Alda-1 is not suitable for use in the clinic owing to its relatively low potency and solubility, this example demonstrates the possibility of testing more clinically suitable analogues using this hiPSC disease model.

### Lysosomal storage disorders

Infantile-onset Pompe disease is an autosomal-recessive glycogen-storage disorder caused by mutations in the *GAA* gene, which encodes the lysosomal enzyme α-glucosidase. Cardiac hypertrophy is frequently detected in patients between 3 and 5 months of age (van der Ploeg and Reuser, 2008), due to accumulation of glycogen in the heart (Thurberg et al., 2009). Similarly, Fabry disease (FD) results in the accumulation of globotriaosylceramide (GL-3) owing to a deficiency in the lysosomal enzyme α-galactosidase A (Zarate and Hopkin, 2008). FD usually develops in adulthood with clinical features including cardiac hypertrophy with diastolic dysfunction, arrhythmia, conduction defects, and myocardial fibrosis (Linhart and Elliott, 2007). The current treatment for both disorders is based on enzyme replacement therapy (ERT) using either recombinant human α-glucosidase (rhGAA) or α-galactosidase A, respectively. However, these treatments are not curative: Pompe patients can develop immunogenic reactions as well as arrhythmias following repeated administration (Kishnani et al., 2009), whereas long-term reduction of GL-3 deposits in FD patients is not observed (Thurberg et al., 2009). To develop improved therapeutic strategies, further understanding of the pathophysiology of these disorders is necessary.

There have been reports of hiPSC models for at least 11 lysosomal storage disorders (Borger et al., 2017). The hiPSC-CMs from patients with the infantile form of Pompe disease exhibited many hallmarks of the disease, including reduced lysosomal α-glucosidase activity, lysosomal glycogen accumulation and lysosome enlargement (Huang et al., 2011; Raval et al., 2015). Likewise, treatment with rhGAA resulted in a significant reduction in glycogen in Pompe disease hiPSC-CMs (Huang et al., 2011). Moreover, treating these hiPSC-CMs with L-carnitine partially rescued some mitochondrial functions, resulting in an increase of oxygen consumption rate that was not observed with the standard treatment, suggesting that this could be a valuable adjunct therapy.

FD hiPSC-CMs also mirrored patient phenotypes, with progressive lysosomal accumulation of GL-3, increased lysosomal storage inclusions and disorganised contractile fibres (Itier et al., 2014). Substrate reduction therapy (SRT) has been proposed as an alternative to ERT to treat FD, with the aim of reducing glycosphingolipid synthesis and therefore decreasing GL-3 levels (Platt and Jeyakumar, 2008). Indeed, SAR 402671, a glucosylceramide-synthase inhibitor, is currently in clinical development for FD (Coutinho et al., 2016). Treating FD hiPSC-CMs with SAR 402671 both prevented GL-3 deposits accumulating and reduced GL-3 levels by more than 50% in FD hiPSC-CMs in which GL-3 had accumulated (Itier et al., 2014), corroborating results obtained using an FD mouse model (Marshall et al., 2010) and highlighting the potential of SRT as an alternative approach for treating the cardiac phenotype of FD.

### Endoplasmic reticulum disorders

By combining hiPSC disease modelling with next-generation sequencing to identify new genetic loci associated with SCD, Devalla et al. (2016) identified two new homozygous loss-of-function mutations in a newly discovered gene that encodes trans-2,3-enoyl-CoA reductase-like (TECRL). These mutations were present in patients from three different families who exhibited characteristics of LQTS and CPVT. However, this disorder is not thought to be a primary channelopathy, because *TECRL* encodes an endoplasmic reticulum (ER) protein that may be involved in lipid metabolism. The clinical phenotype differed according to the mutation, with patients harbouring p.Arg196Gln being diagnosed with LQTS, whereas patients with c.331+1G>A, which causes incorrect protein splicing, were diagnosed with CPVT (Devalla et al., 2016). The hiPSC-CMs derived from a patient with the c.331+1G>A mutation reflected the CPVT phenotype, with abnormalities in calcium handling, including a smaller amplitude and slower decay of cytosolic Ca^2+^ transients. Additionally, prolongation of APD and increased propensity for DADs during catecholaminergic stimulation were observed. As shown with CPVT hPSC-CMs (Itzhaki et al., 2012; Preininger et al., 2016), flecainide reversed the phenotype in the TECRL hiPSC-CMs, although some DADs were still observed (Devalla et al., 2016). Further studies into the exact function of TECRL and its role in calcium homeostasis using these as well as additional hiPSC lines could promote the development of more effective therapies.

### Diabetic cardiomyopathy

Diabetic cardiomyopathy is a long-term complication in type 2 diabetes. It is characterised by structural and functional abnormalities of the myocardium but without coronary artery disease or hypertension (Miki et al., 2013). The underlying pathophysiologic mechanisms are not well understood owing to its multifactorial etiology. Current clinical treatments include glycaemic control, ACE inhibitors and β-blockers. *In vitro* modelling of complex diseases that include an ‘environmental’ factor can be a challenge, but it was recently demonstrated that the cardiac phenotype of diabetic patients could be modelled using hiPSC-CMs (Drawnel et al., 2014), supporting the view that a genetic component contributes to the disease (McCarthy and Zeggini, 2009). Furthermore, when hiPSC-CMs from healthy donors were exposed to a diabetic milieu consisting of glucose, endothelin 1 and cortisol, they developed a cardiomyopathy phenotype that included cellular hypertrophy, increased brain natriuretic peptide release, myofilament disarray, as well as lipid accumulation and peroxidation. To identify potential protective drugs, a 480-compound library was screened; 28 small molecules that prevented diabetic cardiomyopathy were identified. The most effective compounds across all the cellular models were thapsigargin and the voltage-gated Ca^2+^-channel inhibitor fluspirilene (Drawnel et al., 2014). Further studies incorporating *in vivo* testing of this narrower list of effective compounds will provide a stronger base for subsequent clinical development, whereas a more diverse set of hiPSC-CMs derived from type-2 diabetic patients could assist in the delineation of disease subtypes and tailoring of drug treatments.

## Challenges in cardiac disease modelling

Despite the insights that have been gained into multiple cardiac disorders using hPSC-CMs, these models are far from perfect and further developments in cell culturing, measuring functional readouts, and predicting drug responses are still required, as discussed below.

### Immaturity of hPSC-CMs

It is widely acknowledged that a key limitation of hPSC-CMs as disease models is their immaturity. The hPSC-CMs display the typical morphological characteristics of foetal cardiomyocytes (Veerman et al., 2015) and their gene expression profile is also similar to first-trimester gestational stage cardiomyocytes, with several ion-channel- and contractile-protein-encoding genes poorly expressed (van den Berg et al., 2015; Xu et al., 2009; Synnergren et al., 2012). Functionally, this contributes to the immature phenotype of spontaneous contraction, depolarised resting membrane potential (RMP) due to a low or absent inward rectifier K^+^ current (*I*_K1_) and altered Ca^2+^ handling (Lundy et al., 2013; Ma et al., 2011; Sartiani et al., 2007). The conduction velocity in hPSC-CMs is also substantially slower than that of adult cardiomyocytes (Lee et al., 2012). Similar to foetal cardiomyocytes, hPSC-CMs predominantly produce energy through glycolysis, whereas adult cardiomyocytes preferentially generate energy via fatty-acid oxidation (Kim et al., 2013).

Despite their immature phenotype, it has been possible to detect clinically expected characteristics of genetic cardiac disorders using hPSC models. Nonetheless, their sensitivity and accuracy as disease models would be further improved by generating cardiomyocytes that more closely resemble those in adults, because many cardiovascular diseases, such as coronary artery disease and atrial fibrillation, are late onset (Smith and Newton-Cheh, 2015). Most approaches to develop mature hiPSC-CMs aim to mimic the cues that drive heart development *in vivo*. This typically involves long-term culturing of hPSC-CMs to induce morphological changes as well as improve electrophysiological and Ca^2+^ handling (Lundy et al., 2013); however, this is both impractical and costly. Other approaches include co-culture of hPSC-CMs with other cell types also present in the heart, such as endothelial and smooth-muscle cells, and fibroblasts, to increase the resemblance to native myocardium (Tulloch et al., 2011; Giacomelli et al., 2017).

Another tactic is to modify the culture medium. For example, thyroid hormones, such as triiodothyronine, have an important role in heart development (Chattergoon et al., 2012) and have been shown to improve Ca^2+^ handling, bioenergetics and contractile force in hPSC-CMs (Ribeiro et al., 2015; Yang et al., 2014). Indeed triiodothyronine in combination with IGF-1 and the glucocorticoid analogue dexamethasone revealed a contractile-force defect in an HCM hiPSC-CM model that was not detected in medium without these components (Birket et al., 2015a). Similarly, the phenotypes of diabetic cardiomyopathy and ACM could be detected by metabolically maturing the hPSC-CMs through supplementing the medium with fatty acids and insulin or a lipogenic cocktail (Drawnel et al., 2014; Kim et al., 2013).

Altering the extracellular matrix surrounding hPSC-CMs can also increase maturity, with improvements in contractility, electrophysiology, sarcomeric length and mitochondrial function reported (Chun et al., 2015; Patel et al., 2015; Zhang et al., 2012). Likewise, modulating the stiffness of the substrate on which hPSC-CMs are plated can influence contractility, as well as the expression of different sarcomeric protein isoforms (Hazeltine et al., 2012; Weber et al., 2016), whereas forcing the hPSC-CMs to align and elongate using pre-patterned structures improved their maturation based on faster Ca^2+^ kinetics (Rao et al., 2013). Using these methods, impaired sarcomere assembly and contractility could be detected in BTHS hiPSC-CMs (Wang et al., 2014b). Cyclic stretch and strain of hPSC-CMs, either mechanically or by electrical-field stimulation, has also generated more mature cardiomyocytes both structurally and functionally (Chan et al., 2013; Hirt et al., 2014b; Kensah et al., 2013; Nunes et al., 2013; Tulloch et al., 2011). Pacing increased the expression of *KCNJ2*, which can lead to increased *I*_K1_ and lower RMP (Mihic et al., 2014). Similarly, adenoviral overexpression of *KCNJ2* in hESC-CMs hyperpolarised the RMP and resulted in loss of automaticity (Lieu et al., 2013). Indeed, adenovirus-mediated overexpression of *KCNJ2* was recently used to generate more mature hiPSC-CMs to study the arrhythmia mechanism of an LQT9 *CAV3* mutation (Vaidyanathan et al., 2016). Manipulation of the RMP can also be achieved *in silico* by dynamic patch clamp (Bett et al., 2013). By artificially injecting *I*_K1_ into hiPSC-CMs, the resulting RMP, upstroke velocity and amplitude are more similar to that of adult ventricular cardiomyocytes. This approach improved the ability to model Na^+^-channel mutations (Veerman et al., 2016) and even to artificially model *KCNJ2* mutations responsible for Andersen-Tawil syndrome type 1 and short QT syndrome type 3 (Meijer van Putten et al., 2015).

It is apparent that a combination of different strategies will be required to generate hPSC-CMs with a more mature phenotype. Whether hPSC-CMs can reach the same of level of maturity as adult cardiomyocytes in experimentally facile formats remains uncertain. Regardless, any advances made will likely improve the sensitivity of the readouts for hPSC-CM disease models.

### Variability between hPSC-CM lines

Another aspect to consider when using patient hiPSCs as disease models is the most suitable control. Genetic differences (i.e. single-nucleotide polymorphisms in the gene of interest or genetic mutations in genetic modifiers) could exacerbate or even mask the disease phenotype when comparing patient hiPSCs to an unrelated hiPSC line. Even between different control hPSC-CMs, the electrophysiological properties are markedly variable (Sala et al., 2016a). A solution is to use gene targeting to produce isogenic cell lines differing only at the mutation or genetic loci of interest (Merkle and Eggan, 2013). Recent developments in endonuclease-based gene-editing systems, in particular CRISPR/Cas9 (Cong et al., 2013; Jinek et al., 2012), have made it significantly easier to correct genetic defects. It is likely that this approach will complement the more traditional method of recruiting patients to generate hiPSC disease lines, in particular when evaluating new therapeutic compounds (Sala et al., 2016b). However, the frequency of endonuclease-induced off-target mutations and the influence of clonal heterogeneity on the disease phenotype are issues that still require further investigation.

### Directed differentiation to different cardiac cell types

To date, most of the established differentiation protocols generate ventricular-like cardiomyocytes (Mummery et al., 2012) and so most disease modelling studies have focussed on the cell-autonomous ventricular aspects of the disease. However, many channelopathies can also affect other cardiomyocyte subtypes, such as nodal and Purkinje cardiomyocytes in cardiac-conduction disorders and atrial cardiomyocytes in atrial fibrillation (Amin et al., 2010). Several methods have been reported to improve the generation of different cardiomyocyte subtypes using either directed differentiation protocols or through purification (see Box 3). It will be interesting to determine whether subtype-specific disease-causing differences can be detected. Additionally, some diseases, such as ACM and BrS, are known to have ventricular-specific (right versus left) features (Corrado et al., 2016). Developing technologies to generate and distinguish the type of ventricular hPSC-CMs will enable investigations into the chamber-specific characteristics of the disease.

**Box 3. Generating chamber-specific cardiomyocytes from hPSCs**

Now that many of the challenges associated with efficiently generating cardiomyocytes appear to have been solved, there is a desire to improve these protocols such that the hiPSC-CMs can be directed to form cardiomyocytes with specific chamber-like features. Indeed, purification and directed differentiation protocols show increasing potential to obtain pure populations of atrial-, pacemaker-, ventricular- and nodal-like subtypes. To date, subtype purification has relied on the generation of genetically modified hiPSC lines containing fluorescent reporters under the control of a cardiomyocyte subtype-specific promoter. These include selecting for ventricular-like hiPSC-CMs by linking either a green or red fluorescent reporter to the myosin light chain 2v (*MLC2v*) promoter (Bizy et al., 2013; Fu et al., 2010; Huber et al., 2007). Similarly, to enrich for atrial-like hiPSC-CMs, an hiPSC line containing a bacterial artificial chromosome reporter construct in which a red fluorescent protein was driven by expression of sarcolipin (SLN) has been reported (Josowitz et al., 2014). Finally, a lentiviral vector containing the proximal *cGATA6* promoter to drive expression of green fluorescent protein has been used to identify nodal-like hESC-CMs (Zhu et al., 2010).

This line was also used to determine that inhibition of neuregulin-1 signalling increased the proportion of nodal-like hESC-CMs in the culture, whereas activation resulted in more ventricular-like cardiomyocytes (Zhu et al., 2010). Other directed differentiation protocols have shown that, by carefully regulating the retinoic acid and/or the BMP and FGF signalling pathways, cardiomyocytes with pacemaker-like characteristics can be generated (Birket et al., 2015b; Protze et al., 2016). Similarly, modulating the retinoic-acid and Wnt signalling pathways during early stages of differentiation led to hESC- and hiPSC-CMs being directed to either an atrial-like or ventricular-like subtype (Devalla et al., 2015; Karakikes et al., 2014; Zhang et al., 2011). Finally, it was recently reported that timed supplementation of the chemical compound 1-ethyl-2-benzimidazolinone (EBIO) increased the number of cardiomyocytes with nodal- and atrial-like phenotypes (Jara-Avaca et al., 2017).

Because the heart also consists of vascular, smooth-muscle and epicardial cells, it is essential that these cell types can be reliably generated from hPSCs to better mimic their *in vivo* function and to study diseases caused by failing communication between these different cells (Passier et al., 2016). Heterotypic cell models (Box 1) are the next step for investigating non-autonomous diseases such as diabetic cardiomyopathy or myocardial infarction. Also, familial cardiac diseases, such as BrS and ACM, can have a non-cardiomyocyte component, with changes to the epicardium believed to contribute to the overall disease phenotype (Corrado et al., 2016). Methods to derive epicardial cells and their derivatives from hPSCs have been developed (Iyer et al., 2015; Witty et al., 2014) and it is anticipated that more complex multicellular culture systems will be developed. Indeed, a 3D-engineered cardiac-tissue model for HCM was recently reported, in which a fixed percentage of cardiomyocytes (75%) was combined with stromal cells (Cashman et al., 2016). Although key aspects of the HCM phenotype were observed, it is unclear whether the stromal cells contributed to this.

### Predicting clinical responses to therapeutic compounds

As highlighted above, there is also tremendous interest in using hiPSC-CM disease models to help predict how individual patients will respond to particular therapies. Although it is clear that patient hiPSC-CMs typically reflect the overall disease characteristics of the donor, how sensitive these models are for detecting individual differences in disease severity or response to drugs is only starting to be investigated. Early reports have so far been promising, with several recent studies demonstrating that the variation in drug responses observed in some patients with primary arrhythmias were also detected in the corresponding hiPSC-CMs (Penttinen et al., 2015; Preininger et al., 2016; Stillitano et al., 2017). Similarly, it was also demonstrated that hiPSC-CMs could also report the predisposition of some breast cancer patients to develop late heart failure after exposure to the chemotherapeutic drug doxorubicin (Burridge et al., 2016). However, these studies mainly serve as proof of concept, because relatively few patient hiPSC lines were analysed and the study was conducted retrospectively. Developing cost-effective and automated procedures to not only generate hiPSC lines from a larger cohort of patients but also differentiate these to cardiomyocytes will be crucial to further evaluate the potential of this approach in the development of personalised treatment regimes for individual patients.

## Conclusion

Despite the challenges outlined above, the generation of hiPSCs from patients and the ability to derive cardiomyocytes from these cells have resulted in a paradigm shift in cardiac disease modelling. Although hPSCs are unlikely to completely replace animal or heterologous cell-based model systems, hPSC-CMs have proven to be a powerful platform to model various cardiac disorders. This has led to novel mechanistic insights into disease pathologies and aided understanding of these disorders at the individual patient level. This means that new therapeutic compounds and strategies can be tested on human cardiomyocytes from a range of different hPSC lines, potentially leading to treatments that are tailored for individual patients – the ultimate goal of precision medicine.
